# Göte Turesson’s research legacy to *Hereditas*: from the ecotype concept in plants to the analysis of landraces’ diversity in crops

**DOI:** 10.1186/s41065-020-00159-5

**Published:** 2020-11-07

**Authors:** Rodomiro Ortiz

**Affiliations:** Department of Plant Breeding, Swedish University of Agricultural Sciences, Sundsvagen 10 Box 101, SE 23053 Alnarp, Sweden

**Keywords:** Agamospecies, Breeding, Ecotype, Genecology, Landrace, Population genomics

## Abstract

*Hereditas* began with articles on plants since its first issue in May 1920 (six out of eight) and continued with more original articles (43% of the total of this journal) on plants (of which 72% of those in plants were on crops) until today. In December 1922, the 140-page article *The Genotypical Response of the Plant Species to the Habitat* by evolutionary botanist Göte Turesson (Institute of Genetics, Lund University, Åkarp, Sweden) became available. This publication shows that plant phenology has a genetic basis and may ensue from local adaptation. As a result of this research involving various plant species, Turesson elaborated further in this article his term ecotype “as an ecological sub-unit to cover the product arising as a result of the genotypical response of an ecospecies to a particular habitat.” Although plant articles included in *Hereditas* involved from its beginning, trait inheritance, mutants, linkage analysis, cytology or cytogenetics, and more recently gene mapping and analysis of quantitative trait loci with the aid of DNA markers, among others, since the mid-1980s several publications refer to the population biology of plant landraces, which are locally grown cultivars that evolved over time by adapting to their natural and cultural environment (i.e., agriculture), and that may become isolated from other populations of the same crop. This article provides a briefing about research on plant science in the journal with emphasis on crops, summarizes the legacy to genetics of Göte Turesson, and highlights some landrace diversity research results and their potential for plant breeding.

## Plant and crop science in 100 years of Hereditas

**Fig. 1:**
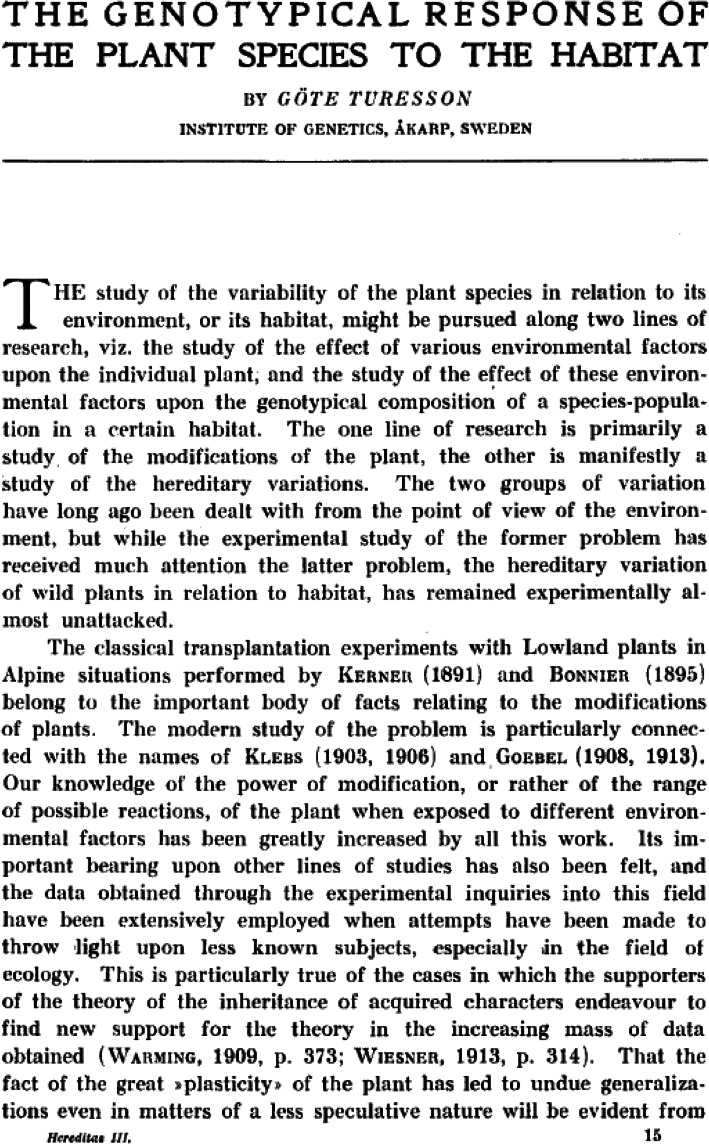
First page of Göte Turensson article in *Hereditas* in which was defined with details the term ecotype

The beginnings of *Hereditas* are tied to plants and the Mendelian Society in Lund (Skåne, Sweden) as indicated by Höglund and Bengtsson [1]. The Mendelian Society in Lund (whose founding was on 10th December 1910) decided in 1920 to launch a journal on genetics aiming a broad audience. The fund raising involved the plant breeding sector that agreed having such a journal could provide knowledge for developing new crops and cultivars. English, French and German were the languages used in the early articles, including those of its first issue in May 1920. There were eight articles, of which ¾ were on plants. The famous geneticist Herman Nilsson-Ehle (1879–1949) [2], then working at at the Institute of Genetics of Lund University in Åkarp (Skåne, Sweden), was the author of the first article focusing on the inheritance of host plant resistance in barley (*Hordeum vulgare*) cultivars to the cyst nematode *Heterodera schachti* [3]. The other five plant articles in the first issue of this journal were on studying the inheritance of characteristics in *Oenothera Lamarckiana*, which in the early years of the 1920s was becoming an important model species for studying genetics and evolution, *Papaver laevigatum*, pea (*Pisum sativum*) and onion (*Allium cepa*); as well as spelt-like bud sports (mutations) in bread wheat (*Triticum aestivum*). Two years later, a 140-page article *The Genotypical Response of the Plant Species to the Habitat* (Fig. 1) by evolutionary botanist Göte Turesson (Institute of Genetics, Lund University, Åkarp, Sweden) brought the term ecotype to define a group of plants resulting from their genotypical response to a specific habitat [4].

**Fig. 2:**
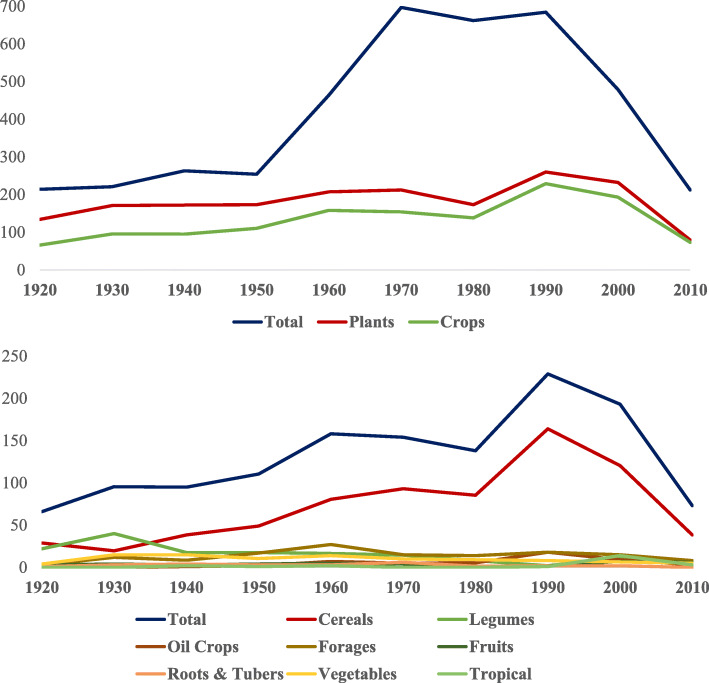
Total number of original articles as well as in wild plant species and crops over decades in the first 100 years of *Hereditas*

Throughout its 100 years, *Hereditas* included, as shown in Fig. 2, in excess of 1800 original research articles on plant species of the wild *Flora Scandinavica* and Fennoscandian grassland species or trees (43% of those included by this journal until now), of which over 1300 (72% of the plant articles) are on many crops. It is worth highlighting that several articles are on mutations in plant breeding, whose research began by Nilsson-Ehle and Åke Gustafsson the 1920s using X-rays and UV radiation on Svalöf’s barley cultivar ‘Gull’. It was during the mid-1930s that valuable mutants for genetic research and breeding were developed through cooperative research between the then Institute of Genetics at Lund University and the Swedish Seed Association [5]. The Institute in Lund became a world-leading center in genetics where later former Nilsson-Ehle’s pupils Arne Müntzing and Albert Levan became research leaders worldwide. The Nordiskt Generesurscenter (NordGen, Alnarp, Sweden) holds today about 11,000 morphological and physiological barley mutants that show changes in their spikes, spikelets, culm length and composition, growth type, kernel development and formation, early heading, awns, leaf blades, pigmentation, chlorophyll development and host plant resistance to powdery mildew caused by the fungus *Blumeria graminis* f. sp. *hordei* [6].

**Fig. 3:**
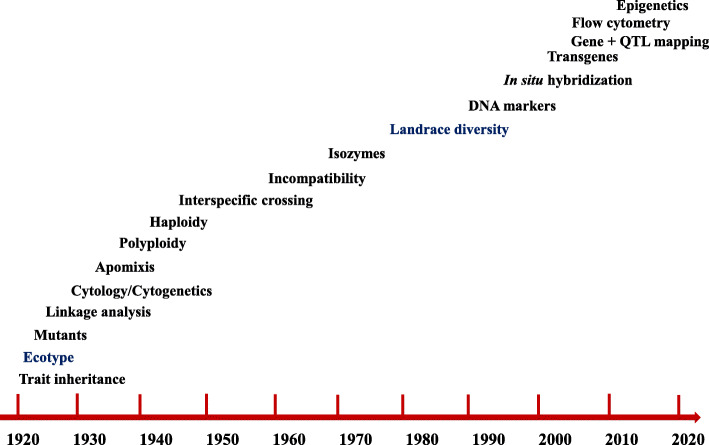
Research topics (as per their first original articles) in crops in the first 100 years of *Hereditas*

Along its history, crop articles in *Hereditas*’ were up to date on developments in genetics, cytology and cytogenetics, as well as cell and molecular biology (Fig. 3). Various articles in this journal were related to the *Allium* test [7], which is used as standard for environmental monitoring. This rapid screening test allows detecting chemicals, contaminants and other pollutants, which are regarded as hazards to the environment. The toxicity is measured by studying root growth inhibition and any adverse effects on the chromosomes of onion. This plant was selected because the ease for its storing, handling and studying both at macroscopic and microscopic levels. The root cells of onion also show the ability to activate pro-mutagens, thus broadening Allium test’s applications.

The in-excess of 1800 plant original articles in *Hereditas* included research on at least 80 domesticated species as well as in their wild relatives. There are 57% original articles on cereals (barley, foxtail and pearl millets –*Setaria italica* and *Pennisetum glaucum* respectively, maize – *Zea mays*, oat – *Avena sativa*, rice – *Oryza sativa*, rye – *Secale cereale*, sorghum – *Sorghum bicolor*, tef – *Eragrostis tef*, triticale – *Triticosecale*, wheat – including both bread and durum or *Triticum turgidum* conv. *durum*), 12% on pulses (Adzuki bean – *Vigna angularis*, bean – *Phaseolus vulgaris*, broad bean – *Vicia faba*, chickpea – *Cicer arietinum*, lupins – *Lupinus* spp., mung bean – *Vicia radiata*, pea), 11% on grass and legume forages (Bahiagrass – *Paspalum* spp., Bermuda grass *– Cynodon dactylon,* bromes – *Bromus* spp.*,* clovers – *Trifolium* spp., cock’s foot – *Dactylis glomerata*, creeping Bentgrass – *Agrostis* spp., fescues – *Festuca* spp., Kentucky’s bluegrass – *Poa pratensis*, lucerne or alfalfa – *Medicago sativa*, ryegrass – *Lolium* spp., signal grass – *Brachiaria* spp., timothy grass – *Phleum* spp.), 8% on vegetables (*Brassica* spp., lettuce – *Lactuca sativa,* onion, peppers – *Capsicum* spp., spinach – *Spinacia oleracea*, table beet – *Beta vulgaris*, tomato – *Solanum lycopersicum*), 4% on oil crops (various *Brassica* species, flax – *Linum usitatissimum*, noug – *Guizotia abyssinica*, poppy seed oil – *Papaver somniferum*, safflower – *Carthamus tinctorius*, sesame – *Sesamum indicum*, sunflower – *Helianthus annuus*, white mustard – *Sinapsis alba*), 3% on temperate fruit (apple – *Malus domestica,* apricot – *Prunus* spp., fig – *Ficus carica*, grape – *Vitis vinifera*, pear – *Pyrus* spp.) and berry crops (currants – *Ribes* spp., *Fragaria* spp., *Rubus* spp., sweet cherry – *Prunus avium*, *Vaccinium* spp. including cranberry – *V. oxycoccos*), 2% on root (cassava – *Manihot esculenta*, cocoyam – *Xanthosoma sagittifolium*, enset – *Ensete ventricosum*, sugar beet – *Beta vulgaris spp. vulgaris* Altissima Group, sweetpotato – *Ipomoea batatas*) and tuber (amochi – *Arisaema schimperianum*, potato – *Solanum tuberosum*) crops, and the remaining between both tropical fruit crops (avocado – *Persea americana*, banana/plantain – *Musa* spp., cacao – *Theobrema cacao*, *Passiflora* spp., pineapple – *Ananas comosus*, watermelon – *Citrillus lanatus*) and aromatic, fibre, fuel, medicinal or recreational plants (e.g. coffee – *Coffea arabica*, cotton – *Gossypium* spp., hop – *Humulus lupulus*, jute – *Corchorus* spp., kenaf – *Hibiscus cannabinus*, *Phalaris* spp., tea – *Camellia sinensis*, switchgrass – *Panicum virgatum*, tobacco – *Nicotiana* spp.).

## Gote Turesson’s ecotype, genecology and agamospecies concepts

The evolutionary botanist Göte Turesson (Malmö, Sweden 6 April 1892 – Uppsala, Sweden 30 December 1970) introduced the concept of ecotype (based on his research of 20 species from 13 genera) while finishing his PhD education at Lund. Turesson understood that the characteristics in a population from natural habitats are both adaptive and hereditary stable. It is worth highlighting that an ecotype does not have a taxonomic rank because individuals from one population are capable of interbreeding with those coming from other population(s), irrespective of the genetic divergence among them. Ecotypes may vary gradually due to adaptive changes; i.e., a cline or the measurable gradient of variation for a characteristic across a geographical range of a species. For example, wild emmer (*Triticum diccocoides*) types showing early heading seems to adapt well in warm and dry sites while those with late heading adapt better in cool and humid sites [8]. Vernalization and earliness are related to heading date in this plant, which highlights the importance of both characteristics for eco-geographical adaptation in this wild relative of wheat. Turensson’s research also demonstrates the genetic basis of periodic cycle events or phenology in plants. Further analysis with DNA markers shows that wild emmer diversity is adaptive due to natural selection and may be influenced by ecological factors [9]. Natural selection was also advocated to explain phenotypic trait variability when comparing wild barley (*Hordeum spontaneum*) among core and peripheral populations from Israel and Turkmenistan [10]. Likewise, allozyme variation was noted to vary and show genetic structuring in populations of the wild grass *Elymus caninus* from Denmark, Finland, Iceland, Italy, Norway, Sweden, Russia, China and Pakistan according to their provenance [11]. This sample of research findings vindicates Turesson’s concept of ecotypes in evolution and the role of natural selection in plant adaptation. A very recent resequencing of 1506 sunflower accessions of non-recombining haplotype blocks associated with many ecologically relevant traits, as soils and climates [12] supports ecotypic differentiation. Divergent haplotype blocks often associated with structural variants maintain adaptive alleles together.

Turesson defined what became known as genecology [13], which refers to the study of genetic variation of the population distribution in a defined environment of both species and communities. In his view, the study of the hereditary variation within species in relation to the habitat was necessary because ecology, until then, did not realize the importance of such variation. His work clearly shows that differentiation among plant populations depends on their genetics rather than on plasticity or the phenotypic changes occurring as a response to the environments where they grow [14]. His research highlights, therefore, that genotype rather than morphology or habitat explains why a plant adapts to an environment [15]. Turesson further argued that the climate affects biotype distribution within a species, as well as that different climatic regions may include genotypically distinct biotype groups [16]. As noted by Heywood [17], Turesson’s genecological hierarchy (which includes ecotypes or ecological races adapted to particular environments, ecospecies consisting of ecotypes, and coenospecies comprising the total ecological potential of a species) was used later by Harlan and de Wet [18] for developing the gene pool concept and its use in plant breeding based on the degree of relatedness between the crop and its wild relatives.

His investigations, which brought new lines of research and have been widely acknowledged elsewhere [19–22], were based mostly on field observations after collecting trips within Sweden, Siberia and North America, among others, and the analysis of empirical data from the experimental garden [23]. Turesson used cock’s foot ecotypes (after collecting them in Siberia during 1927 and testing years later along with two other cultivars at Weibullsholm Plant Breeding Station, near Landskrona, west coast of Sweden) to study agronomic characteristics of “wild type material from which man draws when he ‘improves’ a species population to suit his needs.” [24] His cock’s foot research findings were validated by Stapledon [25], after working with a biotic factor such as “man’s control of his grazing animal.”

Turesson’s first research article explains how the slope exposures influences the distribution of the evergreen conifer Douglas fir (*Pseudotsuga menziesii*) in the arid areas of the state of Washington (USA) [26], where he did his BSc and MSc education. He also brought, after his research on various apomictics including the grass sheep fescue (*Festuca ovina*) and the perennial herbaceous mouse-ear hawkweed (*Pilosella officinarum*), the concept of agamospecies; i.e., natural populations of plant species lacking sexual reproduction whose constituents have a common origin [27]. Turesson was also involved in developing induced tetraploids of red clover (*Trifolium pratense*), which was among the first products of economic importance.

## Landrace diversity and potential for plant breeding

Endashew Bekele, then affiliated with the Department of Genetics at Lund University, wrote in 1985 for *Hereditas* the article *The biology of cereal land race populations* [28], in which he described the patterns of variation of barley, durum and bread wheat. His interest was on developing an evolutionary approach for plant genetic resources conservation and utilization. He proposed a hierarchical approach for defining centers of diversity for both composite populations of landraces and their pests. Bekele indicated that landraces vary along a given ecological gradient due to co-adaptation or competition resulting from the effects of both multi-locus genetic organization and environmental factors. The different multi-locus structures co-evolving with those of their pathogens across regions are without doubts of high interest for plant breeding. Indeed, latitudinal clines of host plant resistance and differential selective pressures at different sites owing to their respective ecological conditions were noted among barley and the fungus *Pyrenophora teres* causing net blotch in this crop [29]. This knowledge may further assist on deploying effectively host plant resistance in farming systems. Moreover, as noted by Dwivedi et al. in a recent up to date review on the subject [30], landraces’ genes provide means for both increasing edible yields and improving adaptation to stress-prone sites. Hence, it was not surprising to read in *Hereditas* several research articles since the mid-1980s about genetic differentiation among composite or landrace populations related to agro-ecological zones or geography (Table 1), thus following on and enlarging Turesson’s research as illustrated in above paragraphs. Most of these articles –often written by former PhD students affiliated with either Addis Ababa University (Ethiopia) or the Swedish University of Agricultural Sciences– were on cereals and particularly from Ethiopian gene pools of crops originating or showing a high diversity in this country, e.g. barley, durum and bread wheat, sorghum or tef. These findings give hopes for an evolutionary plant breeding approach, in which highly genetically diverse crop populations are left to the forces of natural selection that favor plants contributing more seed to the next generation than those with low fitness where they grow.

**Table 1:** A sample of composite cross and landrace diversity analysis for crops in *Hereditas*

Crop	Finding	Reference
Barley	Significant geographical differentiation associated with selection pressures among subpopulations in a composite cross (CCXXI) –as shown by isozyme markers– which increases as generations advance	[31]
	Lacking significant differences for phenotypic diversity (except for aleurone color out of six characteristics) either among 10 regions or among altitudes (< 2000 – > 3501) as well as among agro-ecozones but most of the variance attributed to populations in 51 landrace accessions from Ethiopia	[32]
	High morphological variation within 10 regions and altitudes (particularly above 2000 m a.s.l.) in Ethiopia. Clustering of accessions did not show grouping on the basis of regions of origin	[33]
Wheat	The variation of 13 qualitative or quantitative morphological characters of 293 tetraploid and hexaploid wheat landraces diverged from region to region in Ethiopia. Some of these characters had a localized concentration while others lack a clear distribution pattern. Likewise, a clinal variation pattern was noted for host plant resistance to powdery mildew; i.e., increasing resistance frequency from north to south and Arussi-Bale Highlands showing a concentration of intermediate resistance	[34]
Durum wheat	The first axis of a principal component analysis demonstrated that morpho-physiological variation was related to weather variables affecting drought and heat stress in germplasm from Ethiopia and Syrian germplasm, and to maximum temperatures in germplasm from Turkey	[35]
	Spike density was the only characteristic showing significant differences among Ethiopian regions for 27 landrace populations (being lax spikes noted frequently in Gojam), while glume color and beak length changed significantly according to altitude	[36]
Sorghum	Compact panicles frequently found in relatively dry regions, whereas loose panicles were widely noted in relatively wet and humid regions, thus showing the adaptive significance of panicle compactness and shape	[37]
	Significant allele frequency differences among 48 accessions from 13 regions of origin and 3 adaptation zones (lowlands, intermediate and highlands) in Ethiopia. A Nei’s unbiased genetic distance resulting dendrogram constructed as well as the biplot of the first two principal components distinguished three regions. Gene flow was high among adaptation zones	[38]
	A higher proportion of Ethiopian landraces sharing similar altitude classes and similar ecosites were grouped together after cluster analysis based on ordinal variables. Panicle compactness and shape contributed relatively more than other characteristics to altitudinal and ecological differentiation, thus showing the adaptive significance of both characteristics	[39]
	The clustering from the analysis of molecular variance on 27 accessions was based on four ethnic groups (Soli, Chikunda, Lozi and Tonga), which are associated with collecting sites in Zambia. Most accessions were thus grouped according to their collecting sites.	[40]
Tef	60 Ethiopian populations (comprising 3000 lines) showed significant regional variation for 10 (59% of total evaluated) quantitative traits. Significant clinal variation among altitudes for only six (35%) of these traits. Such results suggest that peasants across regions grow different agro-pheno-morphic types irrespective of altitude	[41]
Amochi	Analysis of molecular variance –based on 167 amplified fragment length polymorphic loci scored from four primer pair combinations split 70.5, 16.7 and 12.8% of the variability between altitudes, as well as between and within populations, respectively, in this Ethiopian tuberous crop	[42]
Noug	Genetic distances based on microsatellites were smaller between populations of neighboring regions in this Ethiopian oil crop, thus placing the UPGMA clustering the populations from neighboring regions closer than those from farther apart areas, and keeping the contiguity of these regions	[43]

Turesson’s genecology and ecotype concepts also provided the foundation for developing the Focused Identification of Germplasm Strategy (FIGS), which allows identifying genebank accessions bearing target traits according to geographic information and agro-climatic knowledge of their collecting sites. FIGS’ underpinnings are related to the fact that environments influence natural selection, thus shaping the geographical distribution of landraces and crop wild relatives. This sampling approach leads to forming ‘best-bet’ trait-specific subsets of genebank accessions, thus facilitating the finding of adaptive characteristics (and their controlling genes) of interest because the probability of success increases as noted, for example, for host plant resistance to Russian wheat aphid [44], or powdery mildew in wheat [45], or drought tolerance in faba bean [46], among others. Incorporating genomics and phenotyping data into a FIGS approach may facilitate identifying alleles from broad genebank holdings. As noted in maize [47, 48] genomic regions controlling time flowering and grain yield were related to adaptation, and the former associated to altitude, while genetic footprints were defined by regions under selection in wheat [49]. Understanding such landrace diversification assists also on breeding new cultivars sustainably since it provides insights regarding crop evolution across stress-prone environments, and for finding genebank accessions and other germplasm whose allelic diversity may be missing in today’s breeding programs.

## Conclusion

Genebank accessions originate mostly from collecting sites (farms or natural habitats) where they evolved over time, thus showing adaptive traits that were shaped by the selection pressures therein. For example, multi-genic analysis of quantitative traits in maize revealed that flowering time alleles are associated with elevation, which indicated that highland adaptation relates to the flowering time pathway [50]. Population genomics uses today, therefore, a large number of high-density DNA markers that are scored in genebank accessions coming from different sites to identify unusual patterns of variation resulting from selection [51]. In this regard, a global sorghum population was characterized with single nucleotide polymorphisms (SNPs) to study the crop population structure [52]. This research provided insights on the patterns of ancient sorghum diffusion to diverse agro-climatic regions across Africa and Asia, thus reinforcing that both agro-climatic constraints and geographic isolation shaped this process. Furthermore, the coupling of population genomics with quantitative genetics led to uncovering mechanisms causing adaptation, e.g. branch length seems to be an agro-climatic trait because dense panicles lead to high yielding sorghum, whereas grain loss reduces with open panicles under humidity. This knowledge led to testing the hypothesis about the feasibility of predicting phenotypic variation based on the assumption that the association between SNP alleles and landrace collecting site reflects adaptation, which was proved in sorghum landraces interacting with environments under drought or aluminum toxicity [53]. Such genomic signatures of adaptation pave the way for a cost-effective “turbo charging” of genebanks, which was demonstrated after integrating genomic prediction into a wide germplasm assessment with the aim of identifying promising sorghum accessions showing high biomass yield [54]. In this regard, after sequencing of rice’s *Heading date 1* (*Hd1*) gene, Wu et al. [55] found three haplotypes related to flowering time across environments in both landraces and modern cultivars, as well as in weedy populations. As stated by Corrado and Rao [56], such genomic scans may unlock instar-specific diversity that differs from that available in modern cultivars and resulting from human selection. Hence, the legacy to genetics of Göte Turesson translates today to using population genomics for researching both ecotype and landrace diversity, which will lead to an in situ or on farm conservation strategy, and their further use in plant breeding.

## Data Availability

Not applicable.

## Abbreviations

FIGS: Focused Identification of Germplasm Strategy
*Hd1*: *Heading date 1* gene in rice
SNPs: Single nucleotide polymorphisms
